# Release of Titanium Particles After Implantoplasty in the Treatment of Peri-Implantitis: Local and Systemic Implications—An Integrative Systematic Review

**DOI:** 10.3390/jcm14248661

**Published:** 2025-12-06

**Authors:** Maria Belén Rodríguez Alvarez, Esteban Padullés-Roig, Guillermo Cabanes-Gumbau, J. A. Callejas-Cano, Javier Gil

**Affiliations:** 1 Patologia Periimplantaria, EDE, University of La Salle, c/Gaminedes 11, 28023 Madrid, Spain; 2 Departament d’Implantologia, EDE, University of La Salle, c/Gaminedes 11, 28023 Madrid, Spain; 3 Bioinspired Oral Biomaterials and Interfaces, Department Ciencia e Ingenieria de Materiales, Escola Enginyeria Barcelona Est. Universitat Politècnica de Catalunya, Av. Eduard Maristany 16, 08019 Barcelona, Spain; drcallejas@gmail.com

**Keywords:** implantoplasty, titanium particles, peri-implant soft tissue, peri-implantitis, TiO_2_, titanium dioxide

## Abstract

**Background/Objectives:** Implantoplasty is widely applied in the surgical management of peri-implantitis; however, this procedure releases titanium micro- and nanoparticles whose biological relevance remains uncertain. Understanding whether these particles influence peri-implant tissue health or systemic responses is essential for assessing the long-term safety of implantoplasty. To determine whether titanium particles generated during implantoplasty are associated with peri-implantitis, peri-implant tissue changes, or systemic effects. **Methods:** This systematic review followed PRISMA 2020 guidelines. Electronic searches were performed in PubMed, Scopus, and Cochrane Library for studies published between 2015 and 2025. Eligible in vitro, in vivo, observational, and clinical studies evaluated titanium particle release during or after implantoplasty and its local or systemic effects. Study selection and data extraction were conducted independently, and the risk of bias was assessed using RoB 2, ROBINS-I, and AMSTAR 2. Synthesis was qualitative due to heterogeneity. Certainty of evidence was evaluated with GRADE. **Results:** Fourteen studies met the inclusion criteria. Titanium particles ranging from 100 nm to 54 µm were consistently detected in peri-implant tissues, with higher levels in peri-implantitis sites, though without consistent clinical association. In vitro data showed dose-dependent inflammatory cytokine release and decreased osteogenic activity, whereas human studies did not confirm a direct relationship between particles and peri-implantitis or marginal bone loss. Certainty of evidence was generally low. **Conclusions:** Titanium particles generated during implantoplasty are detectable but show no consistent clinical association with peri-implantitis or significant inflammation. Implantoplasty may be applied selectively, although robust long-term clinical studies are still required. No protocol was registered.

## 1. Introduction

Peri-implantitis, one of the most significant complications in oral implantology, is an inflammation of the peri-implant mucosa that occurs alongside detectable bone loss surrounding the implant, compromising its function, aesthetics, and stability. In the absence of a universal consensus, Koldsland’s definition of peri-implantitis is considered the most appropriate, as it incorporates both clinical and radiographic diagnostic parameters, considering bone loss ≥ 2 mm + BOP/SUPP (bleeding on probing/suppuration) with PD (probing depth) ≥ 4 or ≥6 mm [1].

**Figure 1:**
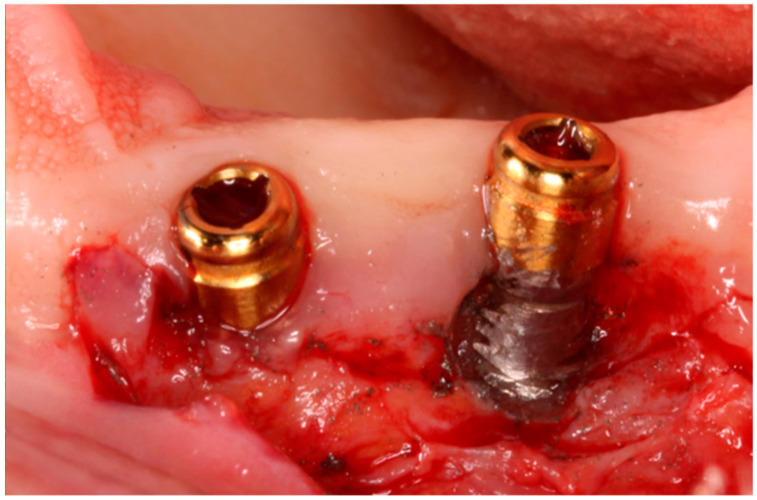
Dental implant treated with implantoplasty and can observe particles in the soft tissue.

**Figure 2:**
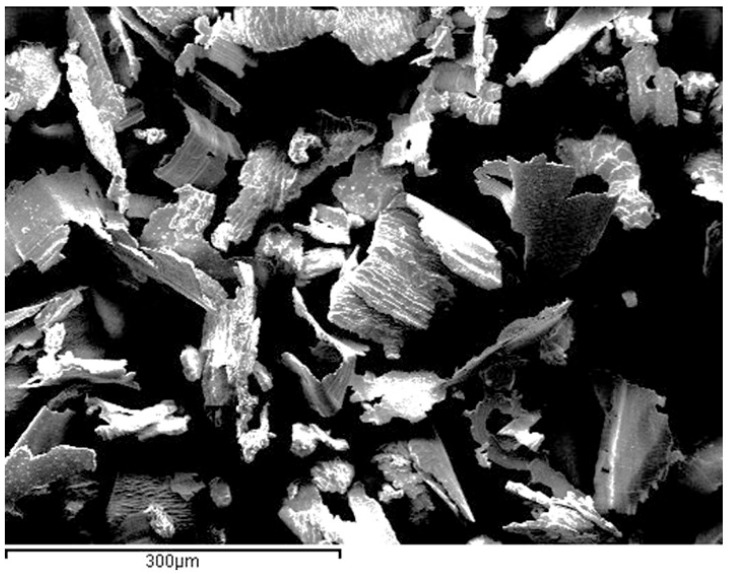
Particles obtained after the implantoplasty observed by scanning electron microscopy.

Although bacterial infection is the main triggering factor, it does not fully explain the pathogenesis; among the causal factors, the release of titanium particles through biotribocorrosion (a synergistic combination of mechanical wear and electrochemical corrosion) has been suggested as a possible inflammatory adjuvant [2]. In this way, it is clinically important to determine whether the presence of these particles is biologically inert or if there is a risk of long-term subclinical effects. The particles in the mouth can be observed after implantoplasty with different sizes in Figure 1. Once extracted and dried of the oral cavity, the particles were observed by scanning electron microscopy. In Figure 2 can be observed the planar morphology due to the effect of the drills.

Kotsakis et al. performed implantoplasty on titanium dental implants in an inflammatory environment simulating that which occurs in the mouth. That is, with salivary fluids and in the absence of oxygen, since inflammation inhibits the presence of oxygen. This is attributed to a response by the immune system which, in the absence of oxygen, kills aerobic bacteria, although anaerobic bacteria, which are the most pathogenic, remain active. Tissues deprived of oxygen may draw oxygen from the titanium oxide layer, partially reducing TiO_2_ under inflammatory conditions, reducing the titanium to pure metal. When the inflammation subsides, oxygen begins to be incorporated, causing the titanium to oxidize, but it does not achieve complete oxidation. Instead, non-stoichiometric mixed oxides are formed, which are cytotoxic [3].

Various studies show that implantoplasty causes a loss in the mechanical properties of dental implants, as it reduces the resistant thickness of the dental implant and the mechanization can produce cracks that promote fatigue. The loss of corrosion resistance of titanium and the increase in ion release into the physiological environment have also been studied. Micrometric particles generated during implantoplasty typically appear dark due to thermal oxidation induced by high-speed instrumentation, to which they have been subjected in order to be removed from the implant, which causes them to oxidize [4].

In clinical practice, implantoplasty, as a treatment for peri-implantitis or as a preventive measure, has demonstrated mechanical efficacy in reducing implant surface roughness and eliminating biofilm. However, its association with particle release has raised concerns in the scientific community and among clinicians. The 2017 Global Workshop on the Classification of Periodontal and Peri-implant Diseases and Conditions noted the existence of evidence suggesting a relationship between titanium particles and peri-implantitis; however, their role as a risk indicator for this condition has yet to be determined more exactly [5].

Although particle concentrations are higher in areas affected by peri-implantitis, there is still no conclusive evidence that their presence directly triggers inflammation or bone loss [6]. In vitro studies have shown that these particles can elicit inflammatory responses [7], yet these findings often fail to correspond with observable clinical outcomes. Current evidence indicates that titanium particle levels may increase substantially within the human body; however, the immunological reactions they provoke remain insufficiently understood and require further systematic investigation. Additionally, several authors have reported that titanium particles are not consistently contained within peri-implant tissues and may disseminate through the bloodstream, accumulating in distant organs and potentially contributing to systemic hypersensitivity reactions and other adverse biological effects [8].

Other studies have begun to focus on the possible effects of the release of titanium particles into the biological environment, not only through implantoplasty but also due to their oral incorporation from other relatively common sources, such as toothpaste, food, or drugs. The European Food Safety Authority [9] issued a warning about the possibility that titanium dioxide (E171), which is used as a food additive, accumulates in tissues, and raises concerns about genotoxicity, despite there being limited concrete clinical evidence.

This review aims to systematically analyze the biological and clinical implications of titanium particles generated during implantoplasty. The specific objectives are as follows: Identify and characterize titanium particles present in peri-implant soft tissue using scanning electron microscopy (SEM) and laser diffraction analyzer to measure the size of the particle and the nitrogen adsorption to determine the specific surface of the particles. Evaluate clinical parameters (probing depth, bleeding on probing, inflammation) in the short, medium, and long term after the procedure. Determine the correlation between the amount of titanium particles and the clinical parameters observed, assessing whether there is any relationship between particle release and the onset of complications such as peri-implantitis or bone loss.

## 2. Materials and Methods

**Figure 3:**
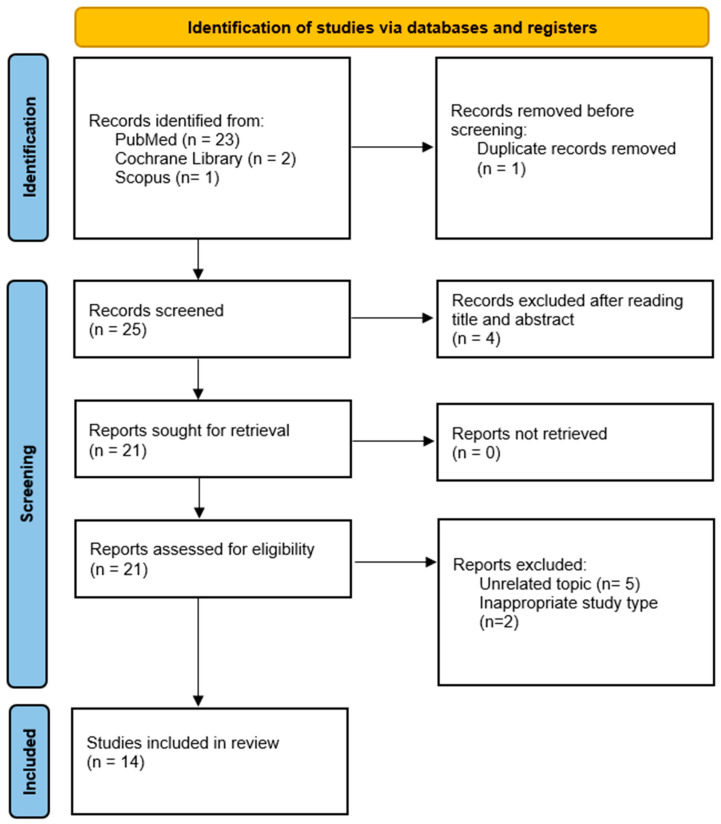
PRISMA flow diagram.

An integrative systematic review has been conducted in accordance with the PRISMA (Preferred Reporting Items of Systematic Reviews and Meta-Analyses) guidelines (Supplementary Materials) [10], in order to identify relevant studies published between 25 May 2015 and 30 May 2025. The PRISMA flow diagram is shown in Figure 3.

A literature review was compiled using the PubMed, Cochrane Library and Scopus databases. For this purpose, advanced Boolean combinations were used, incorporating free terms and MeSH descriptors where applicable: (((implantoplasty) OR (implant surface modification)) AND ((titanium particles) OR (titanium debris) OR (metal particles)) AND ((peri-implant tissues) OR (peri-implant soft tissue) OR (peri-implantitis) OR (inflammation)) AND ((histological response) OR (biological response) OR (inflammatory response) OR (cytotoxicity)))

Before beginning the literature search, the strategy based on PICO (Patient/population, Intervention, comparison, outcome) criteria was taken into account (Table 1).

Eligible studies met the following inclusion criteria: (1) Articles written in English or Spanish; (2) In vivo, In vitro, randomized clinical trial, case–control and observational studies. Systematic reviews may be used for contextual background only; (3) Studies on titanium implants; (4) Studies assessing titanium + article release during or after implantoplasty; (5) Studies evaluating local biological effects (inflammation, cytotoxicity, bone response) or systematic effects. Exclusion criteria: (1) Letters to the editor, editorials, narrative reviews, case reports or anecdotal reports; (2) Studies on implants without documented clinical follow-up; (3) Studies not involving implantoplasty, and (4) Studies on non-titanium implants.

The studies that were ultimately considered eligible addressed the central question: In patients undergoing implantoplasty, is the presence of residual titanium particles in the peri-implant soft tissue, compared to sites without these particles, associated with the absence or presence of peri-implantitis or other clinical complications?

The process has entailed several reviewers working autonomously, examining, comparing, and resolving any discrepancies identified.

Once the studies had been selected according to the defined criteria, the relevant data were extracted using standardized forms. The information collected included:

General characteristics of the study: author, year of publication, study type,

Clinical/experimental context: type of environment evaluated (sites with or without peri-implantitis), duration of follow-up, and presence of implantoplasty procedures.

Detection of titanium particles: techniques used in studies (SEM, TEM, µ-PIXE, ICP-MS, EDX), size range as reported, and concentration quantified in soft or hard tissues.

Primary outcomes included: presence of inflammation (bleeding on probing, probing depth), radiographic bone loss, and cellular/inflammatory response data (cytokines, cell viability, osteogenic activity).

The methodological quality of the studies was assessed using appropriate tools according to the design, such as ROBINS-I for non-randomized studies, RoB 2 for clinical trials, and the AMSTAR 2 tool for systematic reviews. The results were presented in tabular form, which facilitated comparative analysis between screening and selection techniques, study types, and clinical–biological findings. No statistical measures were applied due to heterogeneity; the synthesis was qualitative. Heterogeneity was explored descriptively in the study design, detection technique, attributing differences in implant surfaces and the clinical setting versus in vitro. No sensitivity analyses were performed due to the absence of a combined quantitative synthesis.

The literature search in databases identified 26 articles (Pubmed: 23; Scopus: 1; Cochrane Library: 2). Of these, one duplicate article was discarded, as were four articles that did not meet the criteria (nsufficient data) after reading the title and abstract. Twenty-one full texts were then evaluated individually. Seven full text articles were then excluded with following reasons: inadequate study design, no implantoplasty has been performed, lack of clinical follow-up, or unrelated population. Ultimately, fourteen articles met all criteria and were included in the qualitative synthesis (two systematic reviews, one cross-sectional study, one experimental pilot study, one observational and in vitro study, three observational studies, one case–control study and five in vitro experimental studies). The complete process is illustrated in the PRISMA flowchart.

## 3. Results

The 14 included studies are characterized according to their design, the methods used for particle detection, the model used, and the clinical or in vitro findings in Table 2. Overall, the certainty of the evidence regarding the clinical impact of titanium particles released after implantoplasty is low. In vitro and in vivo studies demonstrate pro-inflammatory potential, cytotoxicity, and reduced osteogenesis, but no conclusive causal association has been established between the presence of particles and the onset of peri-implantitis or other complications. Furthermore, no factors that would increase certainty have been identified.

### Summary of Evidence Synthesis

The main results of these studies are presented below:

**Table 2:** Comparative summary of the included studies.

Author, Year	Study Type	Detection Technique	Main Finding
**Louropaulou et al., 2015 [11]**	Systematic Review	SEM	Particles < 5 µm after implantoplasty; inconclusive inflammation
**Petterson et al., 2017 [12]**	In vitro study	Cytokine analysis	Ti ions form particles that act as secondary stimuli for a proinflammatory reaction
**Suárez-López del Amo et al., 2018 [13]**	Systematic Review	SEM, ICP-MS	Tissue particles; no clinically relevant effects
**Pajarinen et al., 2018 [14]**	In vitro experimental study	SEM, cytokine analysis	Release of proinflammatory mediators such as TNF-α, IL-1β, and IL-6
**Pettersson et al., 2019 [15]**	Cross Sectional Study	SEM,TEM,ICP-MS	Titanium was detected in the peri-implant mucosa, which could aggravate inflammation, but there is no clear clinical correlation
**Berryman et al., 2019 [16]**	Experimental pilot study	SEM-EDS	Over-expression of IL-33 and TGF-B1 in areas with titanium
**Barrak et al., 2020 [17]**	In vitro experimental study	SEM, EDX, ICP-OES	Phagocytosed particles released vanadium with cytotoxicity in fibroblasts, systemic effect not fully understood
**Asa’ad F et al., 2022 [18]**	Observational and In vitro study	SEM, EDX, cytokine análisis, Immunohistochemistry	Titanium particles and ions released at different stages may contribute to peri-implantitis.
**Rakic et al., 2022 [19]**	Case–control study	SEM	No evidence of any body reaction suggestive of direct pathological effects
**Toledano-Serranoba J et al., 2022 [7]**	In vitro experimental study	SEM, cytokine analysis	Increase in TNF-α and IL-1β; decrease in osteogenesis
**Chen and Li, 2023 [6]**	Observational study	SEM, µ-PIXE	More particles in diseased tissues; no clinically significant difference
**Kheder et al., 2023 [20]**	In vitro experimental study	SEM, cytokine analysis	Inflammatory polarization of macrophages
**Platt, A et al., 2023 [21]**	In vitro experimental study	SEM, EDX	Detection of fine particles < 1 µm; no solid clinical data available
**Dionigi et al., 2025 [22]**	Observational study	SEM, ICP-MS	No association was found with clinical peri-implantitis.

Light microscopy (LM), Transmission electron microscopy (TEM), Scanning electron microscopy (SEM), Inductively coupled plasma mass spectrometry (ICP -MS), X-ray microanalysis by proton emissions (µ-PIXE), X-ray spectroscopy (EDX), Inductively coupled plasma optical emission spectrometer (ICP-OES), synchrotron radiation X-ray fluorescence spectroscopy (SRXRF).

The data obtained from the reviewed scientific evidence suggest that the presence of titanium particles in the soft tissues around implants following implantoplasty is not associated with any clinical or inflammatory changes. This finding lends weight to the theory that, even if they are present, these particles may not pose a significant clinical risk under controlled conditions.

**1.**  **Titanium Particles detected:** All studies reported the presence of titanium particles in peri-implant tissue or cell cultures. Most agreed-on ranges from 100 nm to 54 µm, with the most frequent being 1 to 5 µm confirmed by different techniques. Furthermore, particles were identified in both cases with peri-implantitis and in clinically healthy tissues, with no significant intra-individual differences.
**2.**  **Sites analyzed:** Several studies reported higher concentrations of titanium particles in tissue samples with peri-implantitis after implantoplasty, although without demonstrating a direct causal relationship with bone loss or inflammatory progression.
**3.**  **Clinical or experimental evidence:** In vivo studies in which particles have been identified report side effects associated with immune responses, local inflammation, gene expression changes driven by methylation, and alterations in the microbiome; however, these findings have not been directly linked to the development of peri-implantitis. In vitro studies demonstrate a pro-inflammatory reaction characterized by increased levels of pro-inflammatory cytokines and reduced expression of osteogenic markers. Nevertheless, when particles smaller than 1 µm are present, no correlation has been found with compromised peri-implant tissue health or with the progression of peri-implantitis. Moreover, these particles may exert cytotoxic effects.
**4.**  **Biological effects:**
  TNF-α, IL-1β, IL-6
  Macrophage M1 activation
  ROS generation
  Osteoclastogenesis
  Altered fibroblast adhesion
  Epigenetic changes (DNA methylation, miRNA modulation)
**5.**  **Lack of consistent clinical correlation**: despite experimental findings, the clinical studies reviewed found no clinically relevant inflammatory signs in patients with confirmed presence of particles after implantoplasty, especially when adequate plaque control and implant stability were maintained.
**6.**  **Systematic findings**: No strong evidence of systematic toxicity; no study has confirmed systemic dissemination related to implantoplasty.

Several reviewers compared the decisions and resolved their differences through discussion. They consulted a third party when they could not reach a consensus. The GRADE (Grading of Recommendations, Assessment, Development and Evaluation) assessment table is provided in Table 3 and summary of evidence quality (GRADE) for each article in Table 4.

The GRADE assessment of the available evidence showed, overall, low to very low quality in most of the studies analyzed. The systematic reviews included heterogeneous studies with methodological limitations, which led to inconsistency and imprecision. Most in vitro experimental studies presented a moderate risk of bias and high indirectness with respect to the actual clinical situation, reducing confidence in the extrapolation of their findings. Clinical and observational studies, although directly applicable, were often limited by small sample sizes, absence of controls, or lack of adjustment for confounders, which affected precision and risk of bias. In addition, in several cases, the possibility of publication bias was identified due to the small number of studies available. Taken together, these limitations explain the low or very low overall rating of the evidence, indicating that the results should be interpreted with caution and that there is a clear need for better-designed clinical studies with greater statistical power.

## 4. Discussion

Titanium and its alloys (such as titanium–zirconium (Ti-Zr) or titanium–6aluminium–4vanadium (Ti6Al4V)) have been widely used in dental implantology due to their excellent biocompatibility, low modulus of elasticity, high corrosion resistance and osteointegration capacity [23].

Recent advances in implantology have led to a substantial increase in the use of titanium-based components, particularly with the widespread adoption of bone-level dental implants. The compressive forces generated between the rough implant surface and the cortical bone can result in the release of titanium particles into the surrounding environment [24]. A further clinical scenario associated with a significant release of metallic debris is implantoplasty, a procedure commonly employed in the management of peri-implantitis. Implantoplasty involves the mechanical removal of implant threads and textured or modified surfaces to achieve effective decontamination and to create a smoother surface that is less susceptible to bacterial colonization and more amenable to oral hygiene measures [7]. Although the technique has been proposed to improve maintenance when implant threads are exposed, the most recent guidelines for the management of peri-implant diseases conclude that current evidence is insufficient to support definitive recommendations regarding its use.

This procedure induces tribological wear, releasing metal particles whose biological reactivity depends on their size, morphology, and surface charge. Several studies have demonstrated that implantoplasty generates metallic debris capable of accumulating in peri-implant soft tissues at concentrations detectable through techniques such as inductively coupled plasma mass spectrometry and electron microscopy [25,26]. Nonetheless, these findings do not necessarily correlate with clinical deterioration [27]. For example, Dionigi et al. [22] reported no significant differences in inflammatory levels between tissues with detectable titanium particles and those without, in patients both with and without clinical signs of peri-implantitis.

The type of tool used during implantoplasty directly influences the quantity and size of particles produced. Although high-speed rotary tools produce more debris than ultrasonic tools, a standardized protocol to minimize this effect has yet to be established. Therefore, when performing implantoplasty, it is recommended that the type of implant surface, its previous roughness and the surgical technique are considered to reduce the unnecessary release of metal debris [27]. Barrak et al., in their 2020 in vitro study, where they performed implantoplasty with a diamond bur to obtain titanium particles, observed the release of vanadium ions, and concluded that it is necessary to evaluate the materials used in the manufacture of implants and the risks of these individual components of any alloy, taking into account their cellular cytotoxicity [17]. In fact, Padulles et al., in an in vivo study, revealed the presence of tungsten carbide residues on the machined surface as a result of wear of the burr against the titanium surface [28].

Evidence indicates that the surfaces of implants and their restorations are exposed to oral microenvironment conditions, such as low pH, the action of salivary fluids, bacterial load and chemicals that can compromise their titanium dioxide layer, thus initiating corrosion cycles. Implantoplasty studies conducted by Lozano et al. [29] show that the micrometric particles detached from the titanium dental implant exhibit poorer corrosion resistance than the surface of the dental implant after implantoplasty. This is because these particles possess a much higher plastic deformation energy up to fracture. For this reason, the particles appear black due to the formation of mixed oxides. The implantoplasty-treated surface also shows lower corrosion resistance than the control titanium surfaces, owing to its plastic deformation energy (lower than that of the detached particles but higher than that of the control surface). The defective surface finish left by the burs also contributes to this behavior.

Another line of analysis would focus on the morphology, size and composition of the particles, noting that the particles generated during implantoplasty are predominantly smaller than 5 µm [11], which allows them to be phagocytosed by immune cells. However, this phagocytosis does not invariably lead to chronic inflammation, and the studies reviewed have often failed to detect consistent cellular infiltrates in the peri-implant tissues analyzed [13].

The nanometric particles found have a higher surface-to-volume ratio, which increases their chemical reactivity and the possibility of inducing a non-specific immune response [30]. These particles, ranging in size from one nanometer to over 10 µm, have been detected in healthy and pathological peri-implant tissues, raising questions about their actual clinical impact [20].

Experimental studies indicate that titanium particles and ions released at different stages—including implant placement, early healing, and peri-implantitis treatment—may contribute to peri-implant tissue alterations through foreign-body reactions and pro-inflammatory cellular activation [18]. Some authors have proposed that these particles could also influence epigenetic regulation. In vitro, titanium exposure has been associated with modifications in DNA methylation patterns of genes involved in inflammatory surveillance, such as TLR2 and TLR4, potentially enhancing cytokine expression and osteoclastogenic signaling by altering the RANKL/OPG ratio [31]. Fernandes et al. further showed that epigenetic signatures linked to inflammatory activation, including altered promoter methylation and dysregulated microRNAs, are detectable in peri-implant tissues [32]. However, it is important to note that these findings derive largely from general peri-implantitis research rather than implantoplasty-specific models. To date, no study has demonstrated that epigenetic alterations occur directly as a consequence of implantoplasty-generated particles, and this relationship remains hypothetical. Targeted mechanistic studies are needed to clarify whether particle exposure meaningfully contributes to epigenetic modulation in vivo. Mechanical variables and fluorides can affect the amount of metal nanoparticles and ions released by implants and restorations [33]. The bacterial biofilm adhering to titanium not only plays an etiological role in peri-implant inflammation but can also intensify the release of titanium particles through microbial corrosion processes [34].

These data indicate the ability of certain bacterial metabolites, such as organic acids and enzymes, to alter the chemical stability of protective titanium oxide, which increases the release of metal particles and ions into the peri-implant microenvironment. In turn, the accumulation of these particles can amplify the inflammatory response and contribute to the establishment and progression of peri-implantitis, suggesting a bidirectional relationship between bacterial colonization and surface deterioration of the implant [35].

However, the interaction between titanium nanoparticles and microorganisms is complex and not fully understood. While some studies report that biofilm-induced corrosion promotes the release of metal particles and ions [36], other studies have shown that, under certain conditions, titanium nanoparticles can have an antibacterial effect. This effect can be explained by the ability of nanoparticles to alter the integrity of the bacterial membrane, induce oxidative stress by generating reactive oxygen species (ROS), cause modifications in structural and enzymatic proteins, and interact directly with bacterial DNA, affecting its replication and gene expression [6,37].

This scenario of seemingly contradictory findings highlights the complexity of the interaction between bacterial biofilms and titanium particles and underscores the need for further studies, both in vitro and in vivo, to elucidate the molecular mechanisms involved and their clinical relevance in peri-implant health.

Respect to a potential biological susceptibility and proinflammatory cytokines effect on the bone, in 2018, Pajarinen et al., 2019, Petterson et al. and 2023 Kheder et al. in respective in vitro studies, demonstrated that these particles can be phagocytosed by macrophages in the surrounding connective tissue, causing the release of proinflammatory mediators such as TNF-α, IL-1β, and IL-6 [14,15,20]. It can be confirmed that titanium particles act as secondary stimuli for a pro-inflammatory reaction [12]. However, these responses have mainly been detected under conditions of high particle load, suggesting a dose-dependent response that does not always correlate with observable clinical manifestations in humans [38]. Furthermore, an experimental pilot study confirms that most of the inflammatory cells in the peri-implant tissue were chronic inflammatory cells, with overexpression of IL-33 and TGF-B1 in areas where titanium was present, but a relationship between titanium particles and peri-implantitis remains unconfirmed [16]. In turn, A case–control study by Rakic et al. found no evidence of a foreign body reaction. This suggests that titanium particles do not directly cause pathological effects in cases of peri-implantitis. However, the granulation tissue was characterized by intense neovascularization and the presence of a chronic inflammatory infiltrate. This infiltrate was dominated by plasma cells and neutrophils and macrophages [19].

In an exercise to mitigate the risk of peri-implant tissue exposure to titanium particles during implantoplasty, Platt et al., in 2023 [21] conducted an in vitro study on the benefits of performing implantoplasty with and without tissue protection methods, and taking into account several limitations of the study and indications of the pro-inflammatory effect of titanium particles on bone and soft tissue, it can be assumed that the titanium particles generated by implantoplasty are significantly reduced through the use of protective materials, such as rubber dam or bone wax. This finding should be taken into account clinically to avoid iatrogenic inflammatory reactions during implantoplasty.

### 4.1. Other Routes of Exposure to Titanium Dioxide (TiO_2_)

Beyond implantoplasty, there are other sources of exposure to titanium dioxide that should be considered in the context of oral and systemic health. The European Food Safety Authority has issued a warning about the use of the additive E171 in food products due to its potential to accumulate in organs such as the liver and the risk of systemic effects and genotoxicity [9].

Genotoxicity is defined as the ability of a substance to damage a cell’s genetic material, causing mutations that can contribute to the development of neoplastic processes if not repaired. Various genotoxic compounds have been linked to an increased risk of cancer, particularly following chronic and cumulative exposure. Titanium dioxide (TiO_2_) causes DNA damage and increased oxidative stress. It also leads to changes in the expression of genes involved in DNA repair and the inflammatory response, as well as alterations in cell membrane integrity. The evaluation of TiO_2_ nanoparticles and those that can potentially be released during the mechanical manipulation of implants suggests that this risk should be considered in long-term biological safety assessments [39].

Further research has identified the presence of TiO_2_ nanoparticles in toothpaste (which may involve chronic exposure through accidental ingestion, especially in children, although systemic bioavailability is low), medicines and supplements, which may lead to the absorption of nanoparticles through the gastrointestinal mucosa. Following entry into the bloodstream, these particles have the capacity to disseminate and accumulate in various organs, including the liver, spleen, kidneys, lungs, and, albeit to a lesser extent, the brain (where they have been observed to cross the blood–brain barrier), heart (where they have been observed to cause inflammation, cardiac malfunction and heart damage) and testicles. Accumulation in the spleen has been demonstrated to affect immune function, while in the kidney it has the potential to interfere with filtration and excretion mechanisms. Observations have indicated that TiO_2_ is predominantly excreted via feces, with a smaller proportion excreted in urine. This suggests prolonged persistence in the body and the possibility of cumulative effects after repeated exposure [40].

Titanium ion release analyses in physiological media have shown higher ion liberation from dental implants compared to control surfaces; however, the values did not exceed parts per million. Significant changes in ion release were observed when using the Ti6Al4V alloy, where vanadium release increased after 21 days of immersion, reaching tens of parts per million [41,42,43,44]. No clear explanation has yet been found for this selective vanadium release, and this phenomenon requires further study.

Regarding mechanical behavior, static mechanical tests such as bending demonstrated a reduction in fracture strength due to the decreased effective cross-sectional area supporting the dental implant. In fatigue behavior studies, a reduction in the fatigue limit was observed, mainly due to the reduced cross-section and the fact that the number of cycles required for crack nucleation is lower in implants subjected to implantoplasty [45,46,47,48]. This occurs because machining generates small surface fissures or defects that act as stress concentrators, promoting crack initiation. However, an observed effect is that when machining removes the screw threads, the decrease in fatigue life is minor. This occurs because screw threads themselves generate stress concentration in the implant; when they are removed, this concentration disappears, compensating for the section reduction. Therefore, if implantoplasty is to be performed, it is advisable to machine the surface by removing the screw without exceeding this limit.

Biological studies with fibroblastic and osteoblastic cells have shown a reduction in cytocompatibility in commercially pure titanium. According to the standard, a material is considered cytocompatible when cell viability exceeds 70%. However, dental implants made from Ti6Al4V alloy exhibit cytotoxic behavior. For this reason, during implantoplasty procedures, in addition to thorough aspiration, dilution of debris is required, and abundant irrigation with water must be used to dilute potential ion concentrations or residual nano- or microparticles that may remain in the tissues [49,50].

Future research should also investigate the release of titanium particles and corrosion by-products generated during other routine clinical procedures—such as mechanical debridement, ultrasonic instrumentation, or air-powder devices—as these interventions can disrupt the titanium oxide layer and induce biotribocorrosion. Although not addressed in the present review, these procedures may constitute additional sources of micro- and nanoparticle exposure for peri-implant tissues. Systematic evaluation of their biological significance, under standardized experimental and clinical conditions, is essential to contextualize the findings of implantoplasty within a broader framework of titanium surface manipulation.

### 4.2. Limitations

This review presents several limitations. First, implantoplasty is a manual procedure for which no standardized protocols currently exist, and clinicians employ a wide variety of burs and instruments that influence the quantity and characteristics of the particles released into the physiological environment. Additionally, dental implants are manufactured from commercially pure titanium or titanium alloys with differing mechanical properties, such as hardness and toughness, which can further affect the nature of the particles generated. Variability in the amount of pressure applied by clinicians during machining may also contribute to inconsistencies in particle release. Another important limitation lies in the comparison of studies that use diverse characterization techniques with varying detection capacities and sensitivities, potentially leading to differences in reported particle profiles depending on the performance of the analytical equipment. These methodological heterogeneities should be carefully considered when interpreting the findings.

The heterogeneity among the included studies regarding detection methods, sample sizes, clinical criteria, and follow-up duration represents a further significant constraint. Larger, long-term prospective investigations—integrating clinical, histological, and molecular analyses under standardized protocols—are needed to clarify the biological role of titanium particles in peri-implant tissues.

To date, no adverse clinical effects directly attributable to implantoplasty have been demonstrated. However, the biological behavior of nanoparticles that remain embedded in tissues after the procedure remains insufficiently understood. This uncertainty raises concerns regarding their potential local and systemic effects, including long-term consequences related to chemical degradation and cyclic mechanical loading. Further research is required, as the implications for clinical outcomes and potential impacts on distant organs or tissues are still not well established.

Overall, the limitations of this review by subgroups are as follows:

1. **Limitations of in vitro studies**: Many mechanistic findings regarding inflammation, cytotoxicity, or epigenetic modulation are derived from cell cultures. These controlled conditions do not fully replicate the peri-implant microenvironment, limiting extrapolation to clinical scenarios.
2. **Limitations of observational studies:** Most human studies are cross-sectional or of small sample size, restricting causal inference. Variability in implant designs, surgical techniques, and patient factors introduces potential confounding.
3. **Heterogeneity of particle detection techniques:** Techniques such as SEM, TEM, EDX, ICP-MS, and µ-PIXE differ greatly in sensitivity and resolution. Differences in sampling protocols, tissue processing, and reporting thresholds contribute to inconsistent particle quantification.
4. **Indirectness of evidence:** Very few studies assess implantoplasty-generated particles specifically; many examine titanium debris of mixed or unknown origin. Long-term biological behavior of retained nanoparticles remains insufficiently documented, including potential systemic distribution.

These considerations highlight the importance of evaluating cumulative exposure. The combination of peri-implant particle release and continuous environmental or oral exposure suggests a multifactorial risk model [51,52,53]. The integration of existing evidence supports a cumulative-framework perspective, in which the potential effects of titanium should be examined considering both procedure-related local release and systemic exposure through environmental and dietary routes [54]. Nevertheless, direct clinical evidence in humans remains insufficient to establish a clear causal relationship between titanium exposure and adverse health outcomes. Consequently, there is a pressing need for prospective, longitudinal studies that jointly assess the impact of implantoplasty and systemic titanium dioxide exposure [55,56,57,58,59].

### 4.3. Clinical Recommendations

**Selective use of implantoplasty:** Implantoplasty should be indicated in justified clinical contexts, especially in surgical treatment of peri-implantitis, where the reduction in surface roughness may improve periodontal prognosis.

**Controlled technique and standardized protocols**: The use of abundant irrigation systems and torque control during drilling is recommended, as well as the application of post-procedure decontamination protocols to minimize the release of particles.

**Periodic clinical follow-up:** Although no immediate negative effects have been observed, long-term clinical monitoring is suggested in patients treated with implantoplasty, including control of parameters such as probing depth, bleeding on probing and marginal bone changes.

**Caution in susceptible patients:** In patients with a history of immunological disease, titanium allergy, or chronic exposure to metals, a more rigorous risk assessment is recommended before performing procedures that may release metal particles.

**Future research:** Longitudinal clinical studies with prospective design and representative samples, integrating accurate particle quantification with clinical and molecular parameters, are required to confirm the long-term safety of titanium particles in peri-implant tissues.

## 5. Conclusions

The systematic review has revealed two main approaches in the study of implantoplasty. One focuses on particle release, characterizing them as generally less than 10 μm, and on the biological effects that these particles produce. The clinical studies reviewed have failed to establish a direct association between the presence of titanium particles and the development of peri-implantitis, bone loss, or significant tissue inflammation. Although in vitro models show an inflammatory cellular response in the presence of titanium particles, this activation has not been consistently reproduced in clinical practice.

It should be noted that most of the articles included in this review agree on the need to differentiate between observable clinical effects in humans and in vitro cellular responses, as the latter are performed under controlled conditions that do not fully reproduce the microenvironment of peri-implant tissue.

Furthermore, implantoplasty has demonstrated clinical benefits in terms of probe reduction, biofilm control, and bone stabilization, with a low risk of recurrence if accompanied by proper oral hygiene and regular maintenance. These data reinforce the idea that the presence of particles, although detectable, is not a primary pathogenic factor and is not sufficient on its own to cause peri-implant disease. The question of how these titanium particles contribute to the development of peri-implantitis remains a subject of debate, with important issues still to be addressed, such as the long-term behavior of nanoparticles released during implantoplasty and the effects of their chemical and mechanical degradation over time.

## 6. Suggestions for Future Research

Despite the growing amount of evidence, significant knowledge gaps remain regarding the biological and clinical impact of titanium particles released during implantoplasty. Priority areas for future investigation include:

(a) **Long-term controlled clinical trials** to evaluate peri-implant tissue outcomes in patients treated with implantoplasty, as most available studies are cross-sectional, in vitro, or animal-based, limiting causal inference.
(b) **Synergistic interactions with bacterial biofilm,** given that titanium particles may amplify inflammatory responses in contaminated environments. Fretwurst et al. (2018) [53] proposed that particles act as immunomodulatory cofactors rather than primary aetiological agents. New in vitro and in vivo studies combining microbial and particle exposure could clarify this interaction.
(c) **Multicenter studies with harmonized methodologies,** ensuring standardization in particle detection techniques (SEM, EDX, ICP-MS) as well as clinical criteria for inflammation and bone loss.
(d) **Integration of immunological and genetic factors,** since some patients may exhibit a heightened susceptibility to exaggerated responses triggered by metallic nanoparticles.
(e) **Balanced risk–benefit analyses,** addressing both potential systemic risks of titanium exposure and its therapeutic advantages, while considering alternative routes of TiO_2_ intake.
(f) **Long-term systematic evaluations,** aimed at clarifying the real consequences of unintentional exposure to titanium dioxide at the systemic level.

It is important to note that this integrative systematic review was not registered in PROSPERO or other registries, and no protocol was applied to data extraction. All steps were conducted in accordance with the PRISMA 2020 guidelines for transparent reporting.

## Figures and Tables

**Table 1:** PICO strategy.

**Population**	Patients undergoing implantoplasty with titanium particles in peri-implant soft tissue
**Intervention**	Implantoplasty
**Comparison**	Pre-treatment or other mechanical procedures vs. implantoplasty in reference tissue
**Out** **come**	Absence or presence of clinical inflammation, peri-implantitis or systemic effects

**Table 3:** Evaluation of the quality of evidence according to the GRADE (Grading of Recommendations Assessment, Development and Evaluation). RB: Risk of Bias. Inc: Inconsistency. Ind: Indirect. Imp: Imprecision. PB: Publication Bias. Q: Grade of quality.

Author/Year	Study Type	Design Constraints	Main Finding	RB	Inc	Ind	Imp	PB	Other Factors	Q
**Louropoulou et al., 2015 [11]**	Systematic Review	Implantoplasty Studies	Particles < 5 µm; inconclusive inflammation	M	H	L	H	P	Solid structure and comprehensive search, but lacking information on risk assessment and full transparency.	M
**Petterson et al., 2017 [12]**	In vitro study	Study based on cell culture	Ti ions form particles that act as secondary stimuli for a proinflammatory reaction	M	L	M	M	U	Low number of human samples	M
**Suárez et al., 2018 [13]**	Systematic Review	Clinical and in vivo studies	Evidence of frequent release, biological effects still unclear	M	H	L	H	P	High heterogeneity and inconclusive clinical data	L
**Pajarinen et al., 2018 [14]**	Experimental in vitro	Human cells	↑ TNF-α, IL-1β, IL-6	M	L	H	M	U	Indirect clinical relevance, limited extrapolation.	M/L
**Pettersson et al., 2019 [15]**	Cross Sectional Study	Patients, without healthy controls, only periodontitis as a comparison	Ti detected in tissue; no clear clinical correlation	L	ND	L	L	P	Limited evidence due to small sample size, possible publication bias.	L
**Berryman et al., 2019 [16]**	Experimental pilot study	Without control group	Over-expression of IL-33 and TGF-B1 in areas with titanium	M	L	L	M	U	Small sample size	L/M
**Barrak et al., 2020 [17]**	In vitro experimental study	Only cells and culture media, no animal/human model	Implantoplasty releases micro- and nanoparticles; Ti-6Al-4V released V with cytotoxicity in fibroblasts	L	ND	L	L	ND	None	L
**Asa’ad F et al., 2022 [18]**	Observational and In vitro Study	Animals and humans	Released titanium particles/ions may play a pathogenic role in peri-implantitis	M/H	H	M	H	P	Variability in methodology, types of implant surfaces, cleaning techniques, or peri-implantitis treatments, which may alter the risk	L/M
**Rakic et al., 2022 [19]**	Case–control study	Small sample size	No evidence of any body reaction suggestive of direct pathological effects	M	L	L	M	U	Subjective IHC analysis	L/M
**Toledano J et al., 2022 [7]**	In vitro experimental study	Cell cultures	↑ TNF-α, IL-1β; ↓ osteogenesis	L	ND	L	L	ND	Findings that are biologically relevant but not clinically relevant	L
**Chen and Li, 2023 [6]**	Observational study	Patients with peri-implantitis and healthy patients	More particles in diseased tissues; no clinical difference	M	H	L	H	P	Limited sample, useful review but without robust formal methodology	M
**Kheder et al., 2023 [20]**	In vitro experimental study	Human macrophages	Inflammatory polarization	L	L	H	M	U	Indirect clinical relevance	M
**Platt et al., 2023 [21]**	In vitro experimental study	Screening tests	Barriers reduce the release of particles.	L	L	H	M	U	No clinical correlation available	M
**Dionigi et al., 2025 [22]**	Observational study	Patients with multiple implants	Particles present, not associated with peri-implantitis	M	M	L	H	P	Solid design, although limited in size and without adjustments for confusers.	L

L: Low, M: Moderate, H: High, ND: no downgrade, U: unlikely, P: possible.

**Table 4:** Summary of evidence quality (GRADE) for each article.

Author, Year	Summary of Evidence Quality (GRADE)
**Louropoulou et al., 2015 [11]**	The review showed a moderate risk of bias due to a lack of methodological detail and transparency. There was marked heterogeneity between studies, combined with imprecise results due to small sample sizes. Although the evidence was applicable, the possible presence of publication bias reduced overall confidence.
**Petterson et al., 2017 [12]**	The in vitro study presented a moderate risk of bias and limited applicability to the clinical setting. There were no relevant inconsistencies, but the small sample size contributed to imprecision. Publication bias was considered unlikely.
**Suárez et al., 2018 [13]**	The systematic review combined clinical and in vivo studies with high heterogeneity. Methodological limitations and imprecise data reduced confidence, along with possible publication bias.
**Pajarinen et al., 2018 [14]**	The cellular model showed moderate risk of bias and high indirectness with respect to clinical practice. Precision was limited by small experimental sizes, although no inconsistencies were detected and publication bias was unlikely.
**Pettersson et al., 2019 [15]**	The cross-sectional study, although with low risk of bias, was limited by a small sample size and the absence of healthy controls. The evidence was direct and consistent, but limited precision and possible publication bias reduced certainty.
**Berryman et al., 2019 [16]**	The pilot study design without a control group generated a moderate risk of bias and reduced precision. The evidence was applicable and without major inconsistencies, with a low probability of publication bias.
**Barrak et al., 2020 [17]**	The in vitro study had a low risk of bias and adequate precision but limited applicability due to the lack of animal or human models. No inconsistencies or signs of publication bias were detected.
**Asa’ad et al., 2022 [18]**	The combination of observational and experimental studies generated a moderate-to-high risk of bias and high heterogeneity. The evidence was partially indirect and the precision was low, with possible publication bias.
**Rakic et al., 2022 [19]**	The case–control design showed moderate risk of bias, mainly due to the small sample size and the subjective nature of the analysis. The evidence was consistent and applicable, although precision was limited. Publication bias was considered unlikely.
**Toledano et al., 2022 [7]**	The in vitro study had a low risk of bias and good consistency, with acceptable precision. Although indirect with respect to clinical practice, it showed no signs of publication bias.
**Chen and Li, 2023 [6]**	The clinical study presented moderate risk of bias, high inconsistency between patients, and low precision due to limited size. The evidence was direct, but possible publication bias reduced overall certainty.
**Kheder et al., 2023 [20]**	The macrophage model showed low risk of bias and consistent results, but high indirectness limits its applicability. Precision was moderate and publication bias was unlikely.
**Platt et al., 2023 [21]**	The in vitro design showed good experimental control but lacked clinical correlation, which increased indirectness. Consistency was adequate and precision moderate, with no signs of publication bias.
**Dionigi et al., 2025 [22]**	The observational study showed moderate risk of bias due to lack of adjustment for confounders and limited sample size. Although the evidence was direct, moderate inconsistency and imprecision reduced confidence, along with possible publication bias.

## Data Availability

Data extraction sheets and synthesis materials are available from the corresponding author upon reasonable request. The authors can provide details of the research requesting by letter and commenting on their needs.

## Supplementary Materials

The following supporting information can be downloaded at: https://www.mdpi.com/article/10.3390/jcm14248661/s1. In Supplementary Materials there is a PRISMA checklist.
